# Galectin Expression Profiling Identifies Galectin-1 and Galectin-9Δ5 as Prognostic Factors in Stage I/II Non-Small Cell Lung Cancer

**DOI:** 10.1371/journal.pone.0107988

**Published:** 2014-09-26

**Authors:** Iris A. Schulkens, Roy Heusschen, Vivian van den Boogaart, Robert-Jan van Suylen, Anne-Marie C. Dingemans, Arjan W. Griffioen, Victor L. Thijssen

**Affiliations:** 1 Angiogenesis Laboratory, Department of Medical Oncology, VU University medical center, Amsterdam, The Netherlands; 2 Department of Pulmonology, Maastricht University medical center, Maastricht, The Netherlands; 3 GROW - School for oncology and developmental biology, Maastricht University medical center, Maastricht, The Netherlands; 4 Department of Pathology, Maastricht University medical center, Maastricht, The Netherlands; 5 Department of Radiotherapy, VU University medical center, Amsterdam, The Netherlands; Gustave Roussy, France

## Abstract

Approximately 30–40% of the patients with early stage non-small cell lung cancer (NSCLC) will present with recurrent disease within two years of resection. Here, we performed extensive galectin expression profiling in a retrospective study using frozen and paraffin embedded tumor tissues from 87 stage I/II NSCLC patients. Our data show that galectin mRNA expression in NSCLC is confined to galectin-1, -3, -4, -7, -8, and -9. Next to stage, univariable Cox regression analysis identified galectin-1, galectin-9FL and galectin-9Δ5 as possible prognostic markers. Kaplan-Meier survival estimates revealed that overall survival was significantly shorter in patients that express galectin-1 above median levels, i.e., 23.0 (2.9–43.1) vs. 59.9 (47.7–72.1) months (p = 0.020) as well as in patients that express galectin-9Δ5 or galectin-9FL below the median, resp. 59.9 (41.9–75.9) vs. 32.8 (8.7–56.9) months (p = 0.014) or 23.2 (−0.4–46.8) vs. 58.9 (42.9–74.9) months (p = 0.042). All three galectins were also prognostic for disease free survival. Multivariable Cox regression analysis showed that for OS, the most significant prognostic model included stage, age, gal-1 and gal-9Δ5 while the model for DFS included stage, age and gal-9Δ5. In conclusion, the current study confirms the prognostic value of galectin-1 and identifies galectin-9Δ5 as novel potential prognostic markers in early stage NSCLC. These findings could help to identify early stage NSCLC patients that might benefit most from adjuvant chemotherapy.

## Introduction

In non-small cell lung cancer (NSCLC), clinicopathological staging according to the TNM classification is still the main delimiter to classify patients with a distinct outcome. Unfortunately, of the patients diagnosed with early stage disease almost 30% to 40% will present tumor recurrence within two years after surgical resection [1]. Since it has been shown that adjuvant chemotherapy can improve the survival of patients with resected stage II-IIIa NSCLC, identification of early stage patients with poor survival is clinically relevant [1].

Galectins are a protein family of which the members are defined by the presence of a conserved carbohydrate recognition domain [2]. Thus far, fifteen galectins have been identified, eleven of which are also expressed in different human cells and tissues [3], [4]. They exert many different functions, with regulation and fine-tuning of the immune system being the best studied. Consequently, deregulation of galectin expression is frequently associated with an inadequate immune response which contributes to different pathologies, including cancer [5], [6]. In addition, galectins have been found to mediate tumor cell metastasis [7]–[9] and to induce and maintain tumor angiogenesis [10]–[15] which further adds to cancer progression. All this has resulted in the recognition of galectins as diagnostic and prognostic markers in different cancer types, including lung cancer. For example, increased galectin-3 expression has been described as an indicator of poor prognosis in NSCLC patients [16], [17]. Similar observations were reported for galectin-1 expression [16]–[18]. Furthermore, galectin-1 expression is elevated in lung cancer tissue as compared to normal lung [19]. More recently, elevated levels of galectin-1 expression were found to promote lung cancer progression and chemoresistance [20] while increased galectin-4 expression was shown to predict lymph node metastasis in adenocarcinoma of the lung [21]. All these findings illustrate the prognostic potential of galectins in lung cancer. However, whether galectin expression can also be used to distinguish between early stage NSCLC patients with good or bad prognosis has not been well established. Therefore, the objective of this study was to determine whether measurement of galectin mRNA expression could serve as a predictor of clinical outcome in patients with stage I/II NSCLC using a multivariable model.

## Materials and Methods

### Ethical statement

This study was approved by the local internal review board (Maastricht Pathology Tissue Collection, http://www.pathologymumc.nl/research/external-projects/maastricht-pathology-tissue-collection-mptc) and complies with the recommendations guiding physicians in biomedical research involving human subjects as laid down in the Declaration of Helsinki. In accordance with governing ethics, the use of anonymized tissue from the tissue bank did not require specific written consent.

### Patients

The current study included tumor samples of patients with stage I/II NSCLC who underwent an anatomic curative resection at the academic hospital Maastricht between 1994 and 2004 [22]. Exclusion criteria were 1) Previous other malignancy, 2) Development of an unrelated malignancy during a follow-up of at least 4 years, or 3) Neo-adjuvant therapy.

### Specimen characteristics

Resected material was stored at −80°C as part of the Maastricht Pathology Tissue Collection. Only tissues from patients with stage I/II disease and with a tumor area>50% (mean 65.9%, 95%CI: 59.9–71.9), as evaluated in hematoxylin/eosin stained sections by an experienced pathologist (R-JvS), were considered eligible for further investigations.

### Study design

We retrospectively analyzed tumor tissue from stage I/II NSCLC patients who underwent curative resection surgery between 1994 and 2004 at the academic hospital Maastricht. In total, 87 patients were included. The patients received no prior treatment and did not have a history of or develop unrelated malignancies up to 4 years following surgery. The follow-up was at least 5 years during which the patients were examined routinely every 3 months the first 2 years and thereafter every 6 months. Clinical endpoints included overall survival (OS) and disease free survival (DFS). Overall survival was the time in months from the day of surgery until the day of death from any cause. Disease free survival was the time in months from the day of surgery until the day of tumor recurrence, either locoregional or distant. Candidate variables that were considered for inclusion in models included the mRNA expression levels of each galectin (in 2∧-deltaCt), age (in years), stage (I or II), gender, histology (squamous or other), smoking status (former or other). The number of patients included was determined by the availability of tumor samples. Within this sample size, approximately 50 events occurred which allowed the inclusion of 5 variables for multivariable analysis to avoid the risk of over-fitting.

### Cell cultures

The following cell lines were used: A549 human alveolar carcinoma (ATCC CCL-185), H460 human large cell carcinoma (ATCC HTB-177), H1650 human bronchoalveolar carcinoma (ATCC CRL-5883), H1975 human non-small cell lung carcinoma (ATCC CRL-5908), H3255 human non-small cell lung carcinoma (ATCC CRL-2882, discontinued). All cell lines were cultured in RPMI (Invitrogen) supplemented with 10% fetal calf serum, penicillin (50 U/mL) and streptomycin (50 micrograms/mL). Cells were maintained at 37°C and 5% CO_2_ in a humidified incubator.

### RNA isolation and cDNA synthesis

Total RNA was isolated from 10×10 µm thick frozen tissue sections or from cultured cells using the RNeasy kit (Qiagen) according to the manufacturer's instructions. In case of tumor tissue, an additional section was taken before and after the series of 10 for H/E staining and evaluation of the percentage of tumor area. Genomic DNA contamination was removed by on column DNAse treatment. The concentration and purity of the RNA was analyzed using the NanoDrop ND-1000 (NanoDrop Technologies). Subsequently, cDNA synthesis was performed with the iScript cDNA Synthesis Kit (Biorad) using 0.5 to 1.0 micrograms of total RNA.

### Real-time qPCR

qPCR was performed on an iQ5 Multicolour Real-Time PCR Detection System (BioRad) or the CFX96 (BioRad) using the iQ SYBR Green PCR master mix (BioRad) using 400 nmol/L of the appropriate primers which have been described before [23]. To distinguish between the different galectin-9 splice variants the following primers were used: gal-9FL forward GCAGACAAAAACCTCCCG, gal-9FL reverse CCCAGAGCACAGGTTGATG, gal-9Δ5 forward ATCAGCTTCCAGCCTCCC, gal-9Δ5 reverse CCCAGAGCACAGGTTGATG, gal-9Δ5/6 forward CTACATCAGCTTCCAGACCCA, gal-9Δ5/6 reverse CCCAGAGCACAGGTTGATG. qPCR for these splice variants was performed using Sensimix (Quantace) at Tm = 61°C. All primers were synthesized by Eurogentec.

### Western blot

Western blot was performed according to standard protocols. In brief, 5 to 10 10 µm thick crysections were suspended in 60 µL Laemlli sample buffer (Biorad) supplemented with 1∶20 β-mercapto-ethanol. Samples were boiled for 5 minutes and immediately separated by gel electrophoresis on a 15% polyacrylamide gel and transferred onto PVDF membranes (Millipore). Membranes were blocked with Oddyssey blocking buffer (LI-COR Biosciences) for 1 hour and incubated overnight at 4°C with either rabbit anti-galectin-1 antibody (Peprotech) or goat anti-galectin-9 antibody (R&D systems). Loading of the gels was checked by α-actin detection using mouse anti-α-actin (1∶10000, MP Biomedicals). The membranes were washed three times with PBS/0.1% tween and subsequently incubated with the appropriate secondary IRDye antibodies (LI-COR Biosciences) at room temperature for 1 hour. Finally, membranes were washed with PBS/0.1% tween and rinsed with PBS after which images were acquired using the Odyssey infrared imaging system (LI-COR Biosciences).

### Immunohistochemistry

Immunohistochemistry for galectin-1 and galectin-9 was performed on paraffin embedded tissue sections according standard procedures using rabbit anti-galectin-1 (1∶500; Peprotech) and goat anti-galectin-9 (1∶500; R&D systems) antibodies of which the specificity was determined before [23]. Staining were visualized using the StreptABComplex/HRP kit (Dako). The sections were counterstained with hematoxylin (Merck), dehydrated, and mounted in Depex (BDH prolabo). Blinded scoring of galectin-1 and galectin-9 was performed on three different compartments of the tumor, i.e. the tumor cells, the tumor stroma, and the tumor endothelial cells. For scoring of galectin-1 and galectin-9 the frequency of staining was determined using the following scale: 0 = no or hardly any cells positive,1 = small fraction of cells positive, 2 = approximately half of the cells positive, 3 = more then half of the cells positive, 4 = all or the majority of cells positive. All stainings were scored by at least two independent persons.

### Statistical analysis

Bivariable Pearson correlation coefficients were calculated to study the relationships between galectin mRNA expression levels (2^−ΔCt^), clinical parameters, and/or immunohistochemical staining scores. To identify the most important predictors for patients' outcome a two-step approach was used. First, univariable associations between OS or DFS with clinical parameters or each galectin were examined using Cox regression analysis. Next, multivariable Cox regression analysis with forward selection was performed on the most significant galectin predictors identified in the univariable analysis, i.e. gal-1 (categorical), gal-9FL(categorical), gal-9Δ5(categorical), together with age (continuous) and stage (categorical), with either OS or DFS as the outcome. The analysis included the Kaplan-Meier survival estimates with the Log rank test were performed to determine median OS or DFS. Median mRNA expression levels were used as cut-off value to divide the patients into a high expression group (above median) and low expression group (below median). Confidence intervals for median survival were calculated according to Bonnet et al. [24]. All statistical computations were performed in SPSS20.0.0.

## Results

### Expression of galectin mRNA in early stage NSCLC and lung cancer cell lines

We performed extensive galectin gene expression analysis in a retrospective study on resected tumor tissues derived from 87 patients diagnosed with early stage (stage I/II) NSCLC. The median age of the patients was 65.5 years (range 37.4–85.5) and follow-up was at least 5 years during which 47 patients (54.0%) presented recurrent disease and 50 patients died (57.5%). The median overall survival (OS), defined as time between day of surgery until day of death, was 48.7 months (95% CI 33.1–64.2 months). Disease free survival (DFS), defined as time between day of surgery until day of locoregional or distant recurrence, was 33.3 months (95% CI 34.8–49.6 months). The overall demographic and standard prognostic variables of the patient group are listed in Table 1.

**Table 1:** Patient characteristics.

**Total number of patients**	87
**Median age (range)**	65.5 (37.4–85.5)
**Gender**	
Male	65 (74.7%)
Female	22 (25.3%)
**Histology**	
Squamous	43 (49.4%)
Adeno	32 (36.8%)
Large cell	12 (13.8%)
**Stage**	
Stage IA	11 (12.6%)
Stage IB	44 (50.6%)
Stage IIA	6 (6.9%)
Stage IIB	26 (29.9%)
**Recurrence**	
Negative	40 (46.0%)
Positive	47 (54.0%)
**Smoke status**	
Never	2 (2.3%)
Current	21 (24.1%)
Former	59 (67.8%)
Unknown	5 (5.7%)
**Median OS** (months; 95% CI)	48.7 (39.6–53.6)
Events (death)	50 (57.5%)
**Median DFS** (months; 95% CI)	33.3 (34.8–49.6)
Events (recurrence)	47 (54.0%)

**Figure 1:**
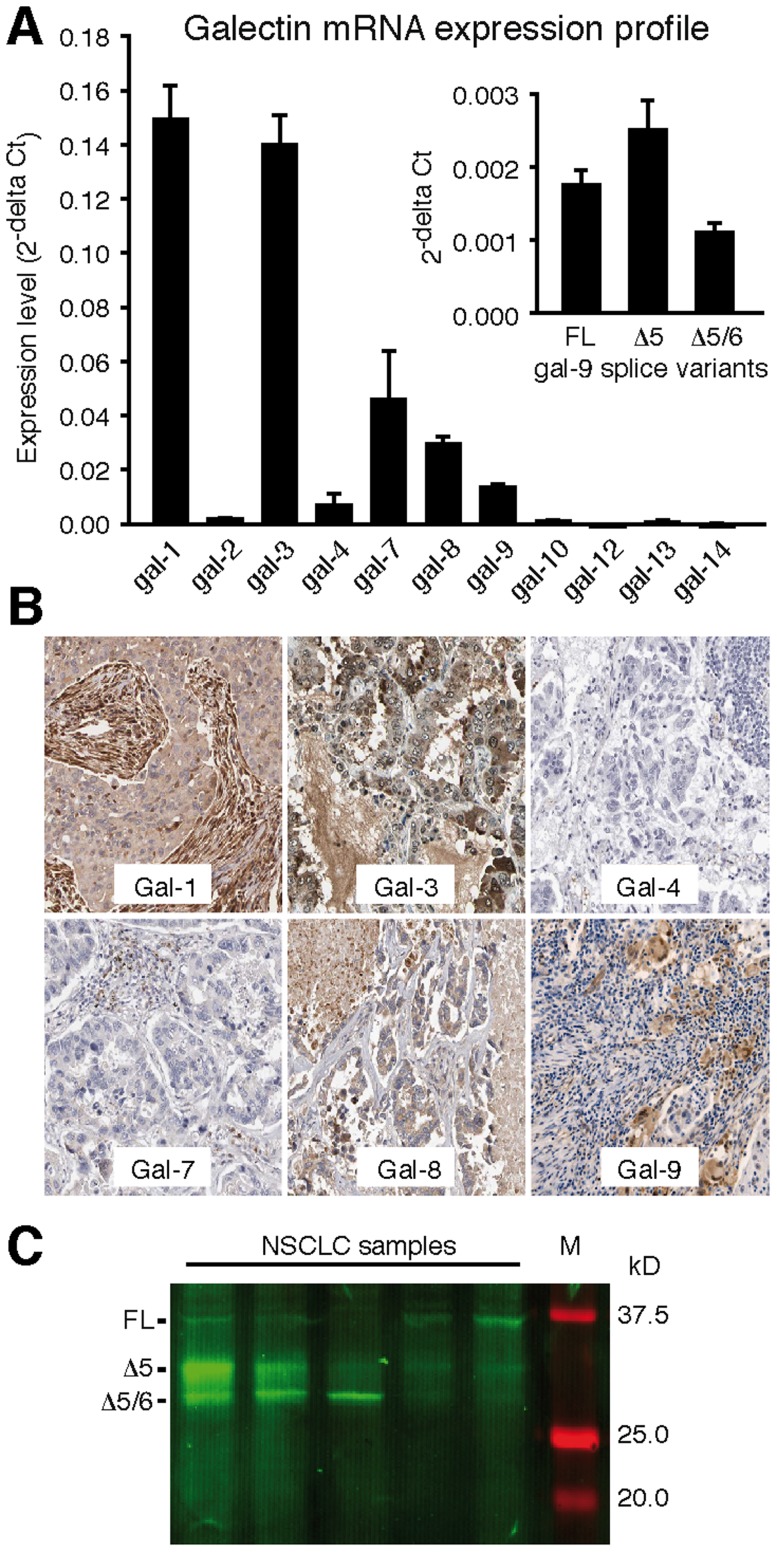
Galectin mRNA expression profile in tumor tissues obtained from early stage non-small cell lung cancer patients (n- = 87) (A). The inset shows the expression of the three galectin-9 splice variants. (B) Images of immunohistochemical staining of the galectins with detectable mRNA expression in NSCLC [27]. (C) Western blot analysis of galectin-9 isoform expression in NSCLC tumor tissue from 5 different patients. Three bands at expected molecular weights of galectin-9FL, galectin-9Δ5 and galectin-9Δ5/6 were observed at varying intensities.

To get insight in the prognostic value of galectin expression in stage I/II NSCLC we first determined which galectins are expressed in NSCLC tumor tissue. qPCR analysis with previously validated primers targeted against all known human galectins [23] revealed that of six galectins, i.e. galectin-1, -3, -4, -7, -8, and -9, mRNA expression could be detected (Figure 1A). Because extensive splicing has been reported for galectin-9 [23], [25], [26], we also determined the mRNA expression of the most common galectin-9 splice variants, i.e. galectin-9 full length (FL), galectin-9 with a deletion of exon 5 (Δ5), and galectin-9 with a deletion of exons 5 and 6 (Δ5/6). All three variants were detectable with gal-9Δ5 as the dominant variant (Figure 1A, inset). Protein expression of the different galectin family members was confirmed by screening immunohistochemical stainings available in the human protein atlas [27] (Figure 1B). Protein expression of the different galectin-9 splice isoforms was further confirmed by Western blot analysis (Figure 1C). Furthermore, expression profiling on different lung cancer cell lines confirmed that expression was confined to galectin-1, -3, -4, -7, -8, and -9 (Figure S1). This corroborates with a study by Lahm et al. who analyzed the expression of multiple galectins in a broad panel of cancer cell lines [28]. All these findings show that galectin-1, galectin-3, and galectin-8 are the most abundantly expressed galectins while the expression of galectin-4, galectin-7, and galectin-9 is relatively low, both in tumor tissues and in different lung cancer cell lines.

### Relationship between galectin mRNA expression and clinical parameters in early stage NSCLC

Analysis of the relationship between the different galectin mRNA expression levels showed a significant positive correlation between total galectin-9 and the specific galectin-9 splice variants, i.e. gal-9FL (r = 0.48), gal-9Δ5 (r = 0.85), and gal-9Δ5/6 (r = 0.52). Within these splice variants there was a significant correlation between gal-9FL and gal-9Δ5 (r = 0.44) as well as between gal-9Δ5 and gal-9Δ5/6 (r = 0.53). Significant correlations were also observed between galectin-3 and gal-9FL (r = 0.34) and between gal-4 and gal-9Δ5/6 (r = 0.31). No significant correlations were found between the mRNA expression levels of the other galectins. Regarding the relationship between galectin mRNA expression and the clinical parameters, i.e. age, stage, gender, histology, and smoking status, we observed a weakly significant correlation between gender and gal-9Δ5 (r = 0.24) and between age and respectively gal-1 (r = 0.26), gal-9 (r = −0.25) and gal-9Δ5 (r = −0.30). No significant correlation between galectin mRNA expression and the remaining parameters, i.e. histology, stage, and smoking status, was found.

### Association between galectin mRNA expression and prognosis in early stage NSCLC

**Figure 2:**
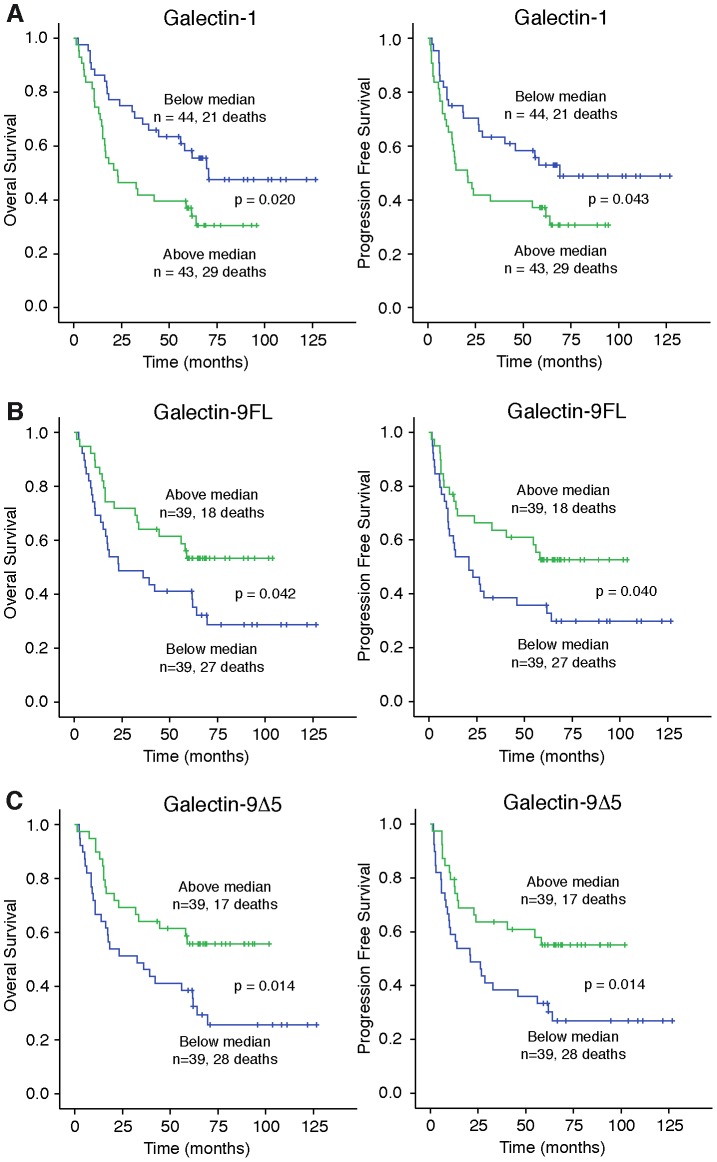
Kaplan-Meier plots of overall survival (OS) or disease free survival (DFS) in early stage NSCLC patients with low (Lo: below median) or high (Hi: above median) expression of galectin-1 (A), galectin-9FL (B) and galectin-9Δ5 (C).

Next, univariable Cox regression analyses were performed to select the markers with the strongest association with OS (Table 2) and DFS (Table 3). This identified stage, gal-1, gal-9FL and gal-9Δ5 as possible prognostic factors for both OS and DFS in early stage NSCLC patients. Subsequently, Kaplan-Meier analyses were used to estimate median OS and DFS in patients that expressed a specific galectin below or above the median mRNA expression level. Patients that expressed galectin-1 above median levels had a significant shorter OS and DFS (Figure 2A and Table 4). The univariable Cox model also identified two splice variants of galectin-9, i.e. gal-9FL and gal-9Δ5, to be possibly associated with both OS and DFS. Indeed, patients with either gal-9FL or gal-9Δ5 expression levels below the median had significant shorter OS as well as shorter DFS (Figure 2B+C and Table 4). None of the other galectins was significantly associated with OS or DFS.

**Table 2:** Univariable analyses of standard clinical variables and galectin mRNA expression levels in relation to overall survival in 87 patients with early stage NSCLC.

	HR	95% CI	p-value
**Age**	1.03	1.00 to 1.06	0.064
**Gender**	0.71	0.37 to 1.40	0.325
**Stage^a^**	1.91	1.09 to 3.35	0.024
**Histology^b^**	0.76	0.44 to 1.33	0.340
**Smoking status^c^**	1.30	0.70 to 2.42	0.402
**Gal1^d^**	1.94	1.10 to 3.41	0.022
**Gal3^d^**	0.60	0.34 to 1.05	0.071
**Gal4^d^**	0.68	0.39 to 1.18	0.170
**Gal7^d^**	0.96	0.53 to 1.74	0.899
**Gal8^d^**	0.78	0.45 to 1.36	0.383
**Gal9^d^**	0.76	0.44 to 1.33	0.333
**Gal9FL^d^**	0.54	0.30 to 0.99	0.045
**Gal9d5^d^**	0.48	0.26 to 0.87	0.016
**Gal9d56^d^**	0.73	0.41 to 1.32	0.304

a) Stage I vs. stage II, ^b)^Squamous vs. others, ^c)^Former smoker vs. others, ^d)^Above vs. below median mRNA expression

**Table 3:** Univariable analyses of standard clinical variables and galectin mRNA expression levels in relation to disease free survival in 87 patients with early stage NSCLC.

	HR	95% CI	p-value
**Age**	1.02	1.02 to 0.99	0.150
**Gender**	0.76	0.76 to 0.39	0.426
**Stage^a^**	1.95	1.95 to 1.11	0.021
**Histology^b^**	0.80	0.80 to 0.46	0.425
**Smoking status^c^**	1.34	1.34 to 0.72	0.357
**Gal1^d^**	1.78	1.78 to 1.01	0.046
**Gal3^d^**	0.58	0.58 to 0.33	0.059
**Gal4^d^**	0.71	0.71 to 0.41	0.231
**Gal7^d^**	0.89	0.89 to 0.49	0.700
**Gal8^d^**	0.79	0.79 to 0.45	0.403
**Gal9^d^**	0.76	0.76 to 0.44	0.336
**Gal9FL^d^**	0.54	0.54 to 0.30	0.044
**Gal9d5^d^**	0.48	0.48 to 0.26	0.017
**Gal9d56^d^**	0.72	0.72 to 0.40	0.283

a) Stage I vs. stage II, ^b)^Squamous vs. others, ^c)^Former smoker vs. others, ^d)^Above vs. below median mRNA expression

**Table 4:** Kaplan-Meier estimates of median OS and DFS in early stage NSCLC patients with galectin expression below or above the median level.

	Median OS (95% CI) in months			Median DFS (95% CI) in months		
	Low^a^ expression	High expression	P	Low^a^ expression	High expression	P
**Gal-1**	59.9 (47.7–72.1)	23.0 (2.9–43.1)	0.020	55.5 (35.7–75.3)	20.8 (−1.35–43.0)	0.043
**Gal-3**	28.0 (5.3–50.6)	58.9 (44.5–73.3)	0.068	16.6 (−5.8–39.0)	58.1 (39.3–76.9)	0.055
**Gal-4**	33.2 (10.1–56.3)	57.5 (41.8–73.1)	0.167	22.4 (−1.9–46.6)	54.7 (31.3–78.1)	0.228
**Gal-7**	57.3 (36.6–78.0)	52.5 (29.5–75.5)	0.899	43.2 (19.9–66.5)	49.7 (25.0–74.4)	0.699
**Gal-8**	40.7 (21.6–59.8)	56.3 (33.9–78.7)	0.382	27.8 (6.4–49.1)	55.0 (30.9–79.1)	0.402
**Gal-9**	42.6 (20.4–64.8)	56.3 (40.85–71.8)	0.331	23.9 (−1.7–49.4)	54.7 (34.7–74.7)	0.334
**Gal-9FL**	23.2 (−0.4–46.8)	58.9 (42.9–74.9)	0.042	20.9 (2.8–39.1)	58.1 (37.4–78.8)	0.040
**Gal-9Δ5**	32.8 (8.7–56.9)	59.9 (41.9–75.9)	0.014	20.9 (−2.5–44.3)	58.1 (36.4–79.8)	0.014
**Gal-9Δ5/6**	39.3 (16.8–61.8)	58.6 (42.0–75.2)	0.302	23.8 (−0.9–48.2)	54.7 (30.0–79.4)	0.280

HR = hazard ratio; CI = confidence interval.

Finally, multivariable Cox regression analysis was performed with forward selection on the most significant factors identified in the univariable analyses, i.e. stage, age, galectin-1 galctin-9FL and galectin-9Δ5. These analyses showed that for OS, the most significant prognostic model included stage, age, gal-1 and gal-9Δ5 while the model for DFS included stage, age and gal-9Δ5 (Table 5).

**Table 5:** Multivariate Cox regression analysis with forward selection.

Overall survival					
Variables in the Equation		HR	95% CI	p-value	overall p-value
Step 1	age	1.05	1.01 to 1.09	0.009	0.008
Step 2	stageI_II	2.41	1.32 to 4.43	0.004	0.001
	age	1.06	1.02 to 1.10	0.002	
Step 3	stageI_II	2.10	1.14 to 3.87	0.018	0.000185
	age	1.06	1.02 to 1.10	0.003	
	gal9d5	0.52	0.28 to 0.97	0.039	
Step 4	stageI_II	2.41	1.30 to 4.49	0.006	0.000099
	age	1.05	1.01 to 1.10	0.008	
	gal1	2.08	1.09 to 3.97	0.026	
	gal9d5	0.46	0.24 to 0.85	0.013	

Disease free survival					
Variables in the Equation		HR	95% CI	p-value	overall p-value
Step 1	gal9d5	0.48	0.26 to 0.88	0.017	0.014
Step 2	age	1.05	1.01 to 1.09	0.016	0.003
	gal9d5	0.47	0.25 to 0.85	0.013	
Step 3	stageI_II	2.08	1.12 to 3.87	0.021	0.000472
	age	1.05	1.01 to 1.10	0.007	
	gal9d5	0.54	0.29 to 1.00	0.051	

HR = hazard ratio; CI = confidence interval.

### Localization and distribution of galectin-1 and galectin-9 protein expression in early stage NSCLC tissue

**Figure 3:**
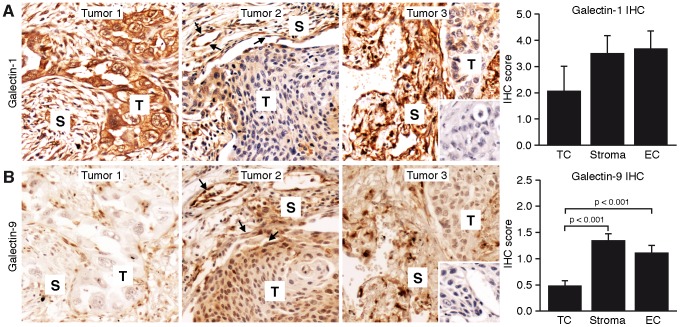
Images of immunohistochemical galectin-1 stainings (A: brown) and galectin-9 stainings (B: brown) of three representative tumors. Staining was observed in tumor cell compartment (T), stromal compartment (S) and in tumor endothelial cells (arrows). The inset on the left shows the isotype control staining. Scale bar = 50 micrometer. The bar graphs showing quantification of IHC scores for galectin-1 and galectin-9 in different tumor compartments.

To get more insight in the localization and distribution of galectin-1 and galectin-9 protein we performed immunohistochemical stainings on a representative subset of tumors (n = 45). Galectin-1 was widely expressed in most tumor tissues. The expression in the tumor cells varied between tumors as well as within tumors, with some tissues showing no positive tumor cells while in other tissues the tumor cells were strongly positive. Most tissues showed positive staining in the stroma as well as in the tumor endothelial cells (Figure 3A). Galectin-9 staining was less prominent as compared to galectin-1. In fact, positive tumor cells were only infrequently observed although some tissues appeared to display a gradient with increasing galectin-9 levels in the tumor cells closer to the stromal tissue (Figure 3B). Both the stroma and the tumor endothelial cells stained positive more frequently (Figure 3B).

Subsequent Pearson correlation analysis of the staining scores showed that there was a significant inverse correlation between the score of galectin-1 and galectin-9 in the tumor cells (corr. coef. −0.36) while there was a positive correlation between galectin-1 and galectin-9 protein score in the tumor endothelial cells (corr. coef. 0.44). However, neither the galectin-1 or galectin-9 protein staining scores were associated with OS or DFS. Furthermore, there was no correlation between IHC scores and mRNA levels.

## Discussion

We evaluated the prognostic significance of galectin mRNA expression in patients with stage I/II non-small cell lung cancer. Univariable Cox regression analyses were used to select a set of the most prognostic clinical parameters and galectins. These were subsequently used in a multivariable analysis to generate a model that could serve to predict OS or DFS in patients with stage I/II NSCLC. The main finding of this study is that for predicting OS, the most significant prognostic model included stage, age, gal-1 and gal-9Δ5 while the model for DFS included stage, age and gal-9Δ5.

Galectins have previously been associated with lung cancer progression [16], [17], [29]. Our observation that patients that express galectin-1 above median levels have a significant shorter overall is in agreement with these studies [16], [17] as well as with studies in other types of cancer [30]. The prognostic value of galectin-1 was confirmed in the multivariable analysis. Galectin-3, which has also been associated with poor disease outcome in lung cancer patients [16], [17], did not reach statistical significance in our patient group. This corroborates with two more recent studies [31], [32]. On the other hand, it has been suggested that cellular localization of galectin-3, i.e. nuclear vs. cytoplasmic might be of prognostic value for recurrence [33]. We only measured galectin-3 mRNA expression levels and did not determine the cellular localization of galectin-3 protein expression in our patient group. Thus, we cannot exclude that these parameters could be of prognostic value in stage I/II NSCLC patients.

A novel finding of the current study was the identification of a specific gal-9 splice variant, i.e. galectin-9Δ5 as a prognostic marker in NSCLC. Using multivariable Cox regression analysis we now observed that low galectin-9Δ5 expression was associated with poor OS and DFS in early stage NSCLC patients. These observations are in line with other reports in which galectin-9 expression was inversely correlated with cancer progression and patient survival in a number of different cancer types, including skin cancer, liver cancer, and breast cancer [34]–[36]. More recently, Jiang et al. identified galectin-9 expression as an independent prognostic factor in a retrospective study on 305 patients with gastric cancer. Again, low galectin-9 expression was associated with poor survival [37].

Galectin-9Δ5 is one of the three most frequently identified galectin-9 variants. These splice variants encode protein isoforms that vary in the length of the linker region between the two CRD domains which affects multimer formation and valency [38], [39]. Previous data suggest that the different galectin-9 isoforms have a diverging role in tumor cells, e.g. they differently affect the adhesion of cells to the ECM and to the endothelium [40]. In general, altered galectin-9 expression has been linked to abnormal cell adhesion, growth and migration [39]. Others have described that galectin-9 can influence cell survival as well as homo- and heterotypic cell aggregation [9], [34], [40]–[43]. Loss of galectin-9 expression could compromise tissue integrity allowing tumor cells to intravasate into circulation and metastasize. Indeed, in breast cancer low galectin-9 expression was a better predictor of distant metastasis compared to lymph node status [42]. Similar observations were made in melanoma and cervical squamous cell carcinoma [34], [44]. However, these effects depend on multiple parameters, including the specific galectin-9 variant, the type of cell and the adhesion matrix component to which the cells bind [45]. Whether and how all these parameters influence lung cancer progression requires further studies. Possibly, galectin-9 can act as a chemoattractant for lung cancer cells, similar as described for eosinophils [46], [47] or endothelial cells [26]. Together with our observation that stromal galectin-9Δ5 expression remains elevated in lung tumors this chemoattracting activity indicates that galectin-9Δ5 might act as a guidance cue for metastatic tumor cells to migrate towards the site of intravasation, i.e. the vasculature. This could promote tumor metastasis especially if loss of galectin-9 in tumor cells results in loss of tissue integrity [45]. Finally, it has been reported that in animal models and cancer patients, tumor cells can release galectin-9 containing exosomes that can induce T-cell apoptosis [48], [49]. Whether tumor endothelial cells also secrete galectin-9 containing exosomes needs to be further investigated, but such a mechanism could contribute to tumor progression by providing a way to escape immune surveillance.

Immunohistochemical assessment of galectin-1 and galectin-9 protein expression showed differences in the localization and distribution within the tumor tissue. These observations are in line with previous findings in different tumors where both galectin-1 and galectin-9 proteins could be detected in different compartments of the tumor, including tumor cells, tumor stroma and tumor endothelial cells [17], [23], [30], [42], [44]. Nevertheless, protein expression had no prognostic value in our patient group. Most likely, this is related to the fact that immunohistochemical staining represents a more qualitative evaluation rather than a quantitative analysis. Thus, actual protein expression levels could not be accurately quantified by IHC staining. Furthermore, no galectin-9 antibodies are available that recognize specific splice variants. This suggests that in case of early stage NSCLC patients, determining galectin mRNA levels is of more value for prognosis estimates as compared to immunohistochemical staining.

The main limitation of the present study is the relatively low sample size of 87 in relation to the large number of parameters that was analyzed. The sample size allowed the inclusion of only 5 covariates in the regression model to minimize the risk of over-fitting. In addition, we only included early stage NSCLC patients. Thus, additional studies using larger patient groups and also including later stages of NSCLC, i.e. stage III/IV, might provide more insight in the prognostic value of galectin mRNA expression profiling.

In summary, extensive galectin expression profiling confirmed the prognostic value of galectin-1 and identified gal-9Δ5 as a potential novel prognostic markers in early stage NSCLC. Identification of such markers is important to identify patients that will benefit from adjuvant chemotherapy. In addition, our findings exemplify the relevance of profiling individual splice variants of galectin-9. It remains to be determined whether splice variant-specific profiling has a similar benefit in other cancer types, including those in which overall galectin-9 expression is a prognostic marker.

## Supporting Information

Figure S1 **Galectin mRNA expression profile in different NSCLC lines.** The inset shows the expression of the three galectin-9 splice variants.
(TIF)
