# Supplementary figures and images for: Galectin Expression Profiling Identifies Galectin-1 and Galectin-9Δ5 as Prognostic Factors in Stage I/II Non-Small Cell Lung Cancer
Source: PLoS One. 2014 Sep 26;9(9):e107988. doi: 10.1371/journal.pone.0107988 (PMC4178059; doi:10.1371/journal.pone.0107988)

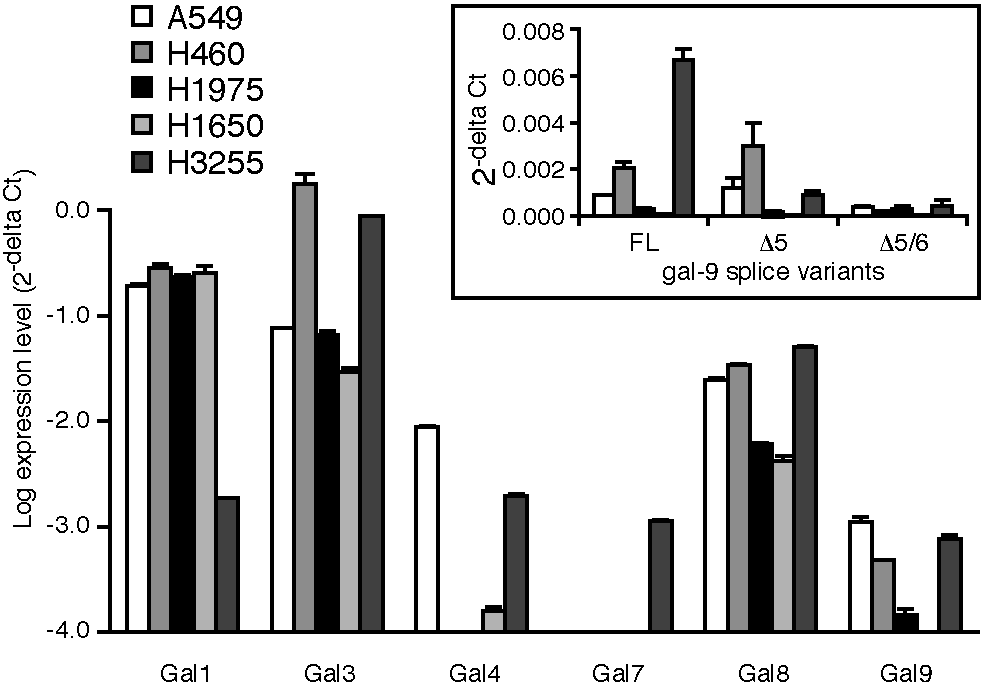

Supplement: Figure S1 — Galectin mRNA expression profile in different NSCLC lines. The inset shows the expression of the three galectin-9 splice variants. (TIF) [file pone.0107988.s001.tif]
